# Molecular characterization of a Minus-C odorant-binding protein from *Cyrtotrachelus buqueti* (Coleoptera: Curculionidae)

**DOI:** 10.3389/fphys.2025.1586738

**Published:** 2025-04-25

**Authors:** Long Liu, Yangdi Li, Hua Yang, Fan Wang, Qiong Huang

**Affiliations:** National Forestry and Grassland Administration Key Laboratory of Forest Resources Conservation and Ecological Safety on the Upper Reaches of the Yangtze River, College of Forestry, Sichuan Agricultural University, Chengdu, China

**Keywords:** *Cyrtotrachelus buqueti*, olfactory, expression pattern, protein expression, competitive binding assay, molecular docking

## Abstract

Odorant-binding proteins (OBPs) are important for insects to discriminate, bind and transport odorants, such as pheromones and host plant volatiles. Herein, the Minus-C OBP (CbuqOBP1) was characterized from *Cyrtotrachelus buqueti*, one of the most important pests in bamboo plantations. *CbuqOBP1* showed significantly higher transcription levels in the adult stage and was most highly expressed in the head of both sexes, the thorax and antenna of the male, indicating that it plays important roles in chemosensory behavior of adults and may also function in other biological processes. Fluorescence competitive binding assays showed that CbuqOBP1 displayed broad binding capabilities and strong affinities to phenol (*K*_i_ = 10.49 μM) and benzothiazole (*K*_i_ = 11.11 μM) among 8 *C. buqueti* volatiles. CbuqOBP1 also showed high binding affinity to the main volatile of the host plant *Neosinocalamus affinis* (linalool, *K*_i_ = 13.41 μM). The docking results indicated that hydrophobic interactions were the prevailing forces between CbuqOBP1 with these three ligands. Additionally, several amino acid residues were significantly overlapped and contributed to the interactions with the ligands. The combined results suggest that CbuqOBP1 may play dual roles in binding volatile compounds from the host plant and the same species and will be helpful to developing new pest-control strategies.

## 1 Introduction

The long-term evolution has endowed insects with a sophisticated and complex olfactory system to accomplish many important physiological behaviors, such as foraging, host seeking, mating, oviposition and predator avoidance (Zhang et al., 2015; Elgar et al., 2018). The olfactory system enables insects to detect and discriminate between a large variety of chemical compounds, even at exceedingly low concentrations (Li et al., 2022). A series of proteins have been found to be involved in the recognition of chemical compounds and triggering of the appropriate chemoelectrical transduction process to unleash behavioral responses, including odorant-binding proteins (OBPs), chemosensory proteins (CSPs), odorant receptors (ORs), gustatory receptors (GRs), ionotropic receptors (IRs), and odorant-degrading enzymes (ODEs) (Leal, 2013).

Among these olfactory-related proteins, OBPs are a family of small, water-soluble proteins consisting of 150–250 amino acids and located in the lymph of the olfactory sensilla. They are commonly accepted to act as the first step in insect olfaction and are responsible for binding and transporting exogenous odorant molecules through the sensillar lymph to the dendrites, where they activate the target odorant receptors (Pelosi et al., 2006). The first insect OBP was identified in the giant moth *Antheraea polyphemus* (Vogt and Riddiford, 1981), and thereafter, an extremely large number of OBP homologs have been isolated and cloned from various insect species of different orders, including Lepidoptera, Coleoptera, Hemiptera, Diptera and Orthoptera (Pelosi et al., 2018; Venthur and Zhou, 2018). OBPs belong to a multigene family, which are divided into four distinct types according to the number of conserved cysteine residues, including Classic OBPs (with six conserved cysteines), Minus-C OBPs (with four conserved cysteines), Plus-C OBPs (with eight conserved cysteines) and Atypical OBPs (with more than eight conserved cysteines) (Venthur et al., 2014; Brito et al., 2016). Due to their extensive gene family and diverse functional roles, OBPs have attracted a research upsurge. In recent years, some progress has been made in understanding the role of Classic OBPs in olfactory systems of Coleoptera, shedding light on their chemosensory mechanisms. For instance, *RferOBP3* and *RferOBP1768* in red palm weevil *Rhynchophorus ferrugineu*s play a role in host plant selection, whereas silencing these two genes reduced the tropism behavior of adult females towards the host plant volatiles (Yuan et al., 2024). It has also been reported that *HoblOBP2*, *4*, *5*, *8*, *9*, and *24* in *Holotrichia oblita* were highly expressed in the antennae and were considered the most important pheromone-binding proteins (PBPs) for recognizing sex pheromones (Qin et al., 2021). Despite these advancements, the olfactory mechanisms in Coleoptera are not as extensively studied as in other insect orders such as Lepidoptera.

Both in Coleoptera and other orders, studies of Minus-C OBPs on molecular identification and functional analysis are rare compared to Classic OBPs. OBP14 from *Apis mellifera* is the first identified Minus-C OBP, which was characterized by only two disulfide bonds formed by four conserved cysteines (Spinelli et al., 2012). It was suggested that Minus-C OBPs might be ancestral proteins, and the absence of one disulfide bond might possess functional relevance, potentially resulting in the formation of a structurally more flexible conformation (Vieira and Rozas, 2011). Recent research indicates that the Minus-C OBP is able to bind host-plant volatiles, implicating a role in host-plant selection (Zhang et al., 2019; Zhang F. et al., 2020; Wang et al., 2024). However, due to the lack of research, binding affinities of Minus-C OBPs to pheromones from other individuals of the same species remain unclear.

The bamboo weevil beetle *Cyrtotrachelus buqueti* belongs to the family Curculionidae within the order Coleoptera. This insect is widely distributed in the southwest of China, coastal areas of Guangdong and Shanghai, and other Southeast Asian countries, such as Vietnam, Myanmar, and Thailand (Wang et al., 2019). It is currently one of the most significant pests in bamboo forests and primarily attacks bamboo shoots by piercing and sucking, as well as laying eggs (Luo et al., 2018a). Leaves damaged by the pest exhibit irregularly shaped bite marks of varying sizes, and entire leaves may be consumed in severe cases, leading to extensive defoliation and significant damage to bamboo plants (Fu et al., 2024). Its infestation rate in dense bamboo forests can reach 50%–90% (Yang et al., 2009). As a significant forestry pest, it has emerged as a major constraint on the development of bamboo forests for papermaking, resulting in substantial economic losses and adversely affecting ecological sustainability (Fu et al., 2024). Currently, the survival of 28 bamboo species is under threat due to its impact, especially in the *Dendrocalamus*, *Bambusa* and *Dendrocalamopsis* genera (Luo et al., 2019). Due to the serious ecological damage and economic losses caused by the pest, the State Forestry Administration of China has listed it as a dangerous forestry pest since 2003 (Luo et al., 2018b).

Understanding the chemical ecology of *C. buqueti* is crucial for developing novel control strategies by interfere with its behaviors such as host location and mating. OBPs are promising molecular targets for these strategies. Although two pheromone binding proteins (PBPs) of *C. buqueti* were identified and their binding affinities have been quantified (Yang et al., 2017b; Liu et al., 2023), our knowledge of OBPs in *C. buqueti* is still insufficient and more investigations are required. And up to date, no Minus-C OBP has been documented in *C. buqueti*. In the present study, the identification and spatial-temporal expression profiles of one Minus-C OBP gene, *CbuqOBP1*, from *C. buqueti* was firstly reported. Recombinant CbuqOBP1 protein was successfully expressed and purified using the bacterial expression system. And then, the function of CbuqOBP1 was tested *via in vitro* fluorescence binding assays with eight volatiles emitted by female adults of *C. buqueti* and one plant volatile from the host plant *Neosinocalamus affinis*. In order to facilitate the understanding of the molecular interaction mechanisms between CbuqOBP1 and ligands, the molecular modeling and molecular docking were carried out. This study aims to provide evidence that CbuqOBP1 are involved in the chemoreception of intraspecific pheromone and provide valuable information for the investigation of the olfactory mechanism in *C. buqueti*.

## 2 Materials and methods

### 2.1 Insect rearing and collection

Pupae of *C. buqueti* were originally obtained from the bamboo planting base (30°13′E, 102°91′N) located on Lushan County, Ya’an City, Sichuan Province, China, and were successfully reared in the laboratory insectary. Larvae and adults were fed with fresh *N. affinis* shoots. Rearing conditions were 25°C ± 1 °C, 12 h light:12 h dark photoperiod and 70% ± 10% relative humidity. For tissue-specific gene expression, adults were sexed after emergence. Various tissues of 3-day-old virgin adults from both sexes were dissected on ice, including antennae, heads (without antennae), thoraxes, abdomens, legs and wings. One sample contained at least five individual tissues. For development stage-specific gene expression, eggs, larvae (mixture of all instar larvae), 3-day-old pupae, 3-day-old female and male adults were collected into Eppendorf tubes (1.5 mL), respectively. Each experiment was carried out in biological triplicate. All samples were promptly frozen in liquid nitrogen and then preserved at a temperature of −80 °C until the isolation of RNA.

### 2.2 Sequence and phylogenetic analysis

The *CbuqOBP1* gene was identified from the *C. buqueti* transcriptome which was constructed and annotated by our laboratory previously (GenBank accession number: SRS1876730) (Yang et al., 2017a). The open reading frame (ORF) of *CbuqOBP1* was determined by using NCBI ORF finder (https://www.ncbi.nlm.nih.gov/orffinder). The N-terminal signal peptide sequence was predicted using SignalP v6.0 (https://www.cbs.dtu.dk/service.php?SignalP). The prediction of the domain architecture was performed using the SMART program (http://smart.embl-heidelberg.de/). The online program tools ProtParam (Expasy) (https://web.expasy.org/protparam/) was used to determine the molecular weight (Mw) and isoelectric point (pI). The amino acid sequence alignment was conducted utilizing the MAFFT method under the auto algorithm and BLOSUM62 scoring matrix (Katoh and Standley, 2013), and the result was visualized using ESPript v3.0 (http://espript.ibcp.fr/ESPript/ESPript/).

The phylogenetic tree of CbuqOBP1 and other coleopteran OBPs was generated by using the maximum likelihood (ML) method. The MAFFT method was used to align these OBPs as described above. Using the ModelFinder integrated in PhyloSuite v1.2.2, the best-fit model of amino acid evolution for selected OBPs was determined based on the Bayesian information criterion (BIC) with default settings (Kalyaanamoorthy et al., 2017; Zhang D. et al., 2020) and the best-fit model was LG + I + G4. Under the best-fit model estimated by ModelFinder, the ML tree of selected coleopteran OBPs was generated using the IQ-TREE in PhyloSuite v1.2.2 with the default parameters (Nguyen et al., 2015). Bootstrap support of the tree branches was estimated by employing 5,000 ultrafast bootstraps (Minh et al., 2013), alongside the application of the Shimodaira–Hasegawa-like approximate likelihood-ratio test (SH-aLRT) with 1,000 replicates (Guindon et al., 2010).

### 2.3 RNA extraction, cDNA synthesis, and quantitative real-time PCR (qPCR)

The MiniBEST Universal RNA Extraction Kit (TaKaRa, Dalian, China) was used to extract total RNA from each sample in accordance with the recommended protocol. The integrity of the total RNA was analyzed by gel electrophoresis. RNA quality (OD_260/280_ > 1.8) and concentration were determined by the DU800 spectrophotometer (Beckman Coulter, CA, United States). First-strand cDNA synthesis was performed using 1 μg of the total RNA with the SweScript RT I first-strand cDNA synthesis kit (Servicebio, Wuhan, China). The final cDNA samples were preserved at −20°C until further analysis.

The qPCR analysis was performed to investigate the expression of *CbuqOBP1* in different stages and tissues of *C. buqueti*. The cDNA was diluted at a ratio of 1:5 in sterilized PCR-grade water, serving as the template for the qPCR analysis. The glyceraldehyde 3-phosphate dehydrogenase (*GAPDH*) of *C. buqueti* (GenBank accession number: KY745870.1) was employed as the internal reference gene. Gene specific antisense primers for *CbuqOBP1* and *GAPDH* were designed using Primer Premier v5.0 and are provided in Supplementary Table S1. The amplification of a single fragment was verified by the melting curve analysis. A standard curve was constructed to determine the amplification efficiencies of the target and reference genes by using the templates diluted into 10-fold series. The qPCR was carried out using TB Green™ Premix Ex Taq™ (Tli RNaseH Plus) Kit (TaKaRa, Dalian, China) in a 25 μL reaction containing 2 μL of sample cDNA, 1 μL each of sense and antisense primers (10 μM), 12.5 μL of TB Green Premix Ex Taq II (Tli RNaseH Plus) and 8.5 μL of sterilized PCR-grade water. Reactions were run according to the following program: an initial denaturation for 30 s at 95°C, followed by 40 cycles of 95°C for 5 s and 60°C for 30 s. Each reaction was run in three technical repeats with three independent biological replicates. The relative levels of gene expression were determined using 2^−ΔΔCT^ method (Livak and Schmittgen, 2001).

### 2.4 Recombinant protein expression and purification

The signal peptide of CbuqOBP1 protein was removed to generate properly folded protein. Using the cDNA as the template, the *CbuqOBP1* sequence that encode the mature protein was amplified by specific primers (Supplementary Table S1). Following the manufacturer’s instruction of the ClonExpress II One Step Cloning Kit (Vazyme, Nanjing, China), the purified PCR products were ligated into the bacterial expression vector pET-28a (+) (Sangon Biotech, Shanghai, China) through homologous recombination. The recombinant plasmid containing the correct insert fragment was then transformed into competent cells of the *Escherichia coli* BL21 (DE3) expression strain. The verified bacterial suspension was inoculated into LB broth supplemented with 50 μg/mL of kanamycin and subsequently incubated at 37°C until the culture reached an optical density value of 0.6–0.8 at 600 nm. The expression of recombinant protein was induced at 20°C overnight by isopropyl β-d-l-thiogalactopyranoside (IPTG) at a final concentration of 0.5 mM. The bacterial cells were harvested by centrifugation at 12,000 rpm for 10 min at 4°C and lysed by sonication on ice. The recombinant protein in the supernatant was purified using Ni-NTA affinity chromatography (Bio-Rad Laboratories Ltd., Shanghai, China) with a gradient concentration of imidazole washing and desalted using Dialysis Membrane (Sangon Biotech, Shanghai, China). The molecular weight and purity of the resulting protein was detected by sodium dodecyl sulfate–polyacrylamide gel electrophoresis (SDS-PAGE) analysis. Using rat anti-His polyclonal antibody (Sangon Biotech, Shanghai, China) as the primary antibody and horseradish peroxidase-conjugated goat anti-rat IgG (Sangon Biotech, Shanghai, China) as the secondary antibody, the purified recombinant protein was also analyzed by Western blot. The concentration of the purified protein was determined by Bradford Protein Assay Kit (Solarbio Science & Technology Co., Ltd., Beijing, China) and was then concentrated to 2 mg/mL using PEG 20,000 and stored at −80°C until use.

### 2.5 Fluorescence competition binding assays

The fluorescence competition binding assays were carried out with a F-7000 fluorescence spectrophotometer (Hitachi, Tokyo, Japan) at room temperature using a 1 cm wide light-path fluorimeter quartz cuvette and 10 nm slits for both excitation and emission. The purified CbuqOBP1 was dissolved in 50 mM Tris–HCl buffer (pH 7.4) to a final concentration of 2 μM, while all ligands used in binding experiments were dissolved in chromatographic methanol to a final concentration of 1 mM.

*N*-phenyl-1-naphthylamine (1-NPN) (Aladdin Biochemical Technology Co., LTD., Shanghai, China) was used as the fluorescent probe. The probe was excited at 337 nm and emission spectra were recorded between 350 and 550 nm. The affinity of 1-NPN to CbuqOBP1 was measured by titrating the protein with aliquots of 1-NPN at final concentrations ranging from 2 to 40 μM, and the corresponding fluorescence intensities were recorded. Assuming that 1-NPN binds to the protein at a 1:1 ratio and that the protein was 100% active, a Scatchard plot analysis of the binding data was performed to calculate the dissociation constant (*K*_d_) of CbuqOBP1 with 1-NPN.

Previous behavioral studies showed that the volatiles emitted by female adults of *C. buqueti* exhibited a robust attractant effect on males (Yang et al., 2010; Mang et al., 2012). Additionally, our previous studies indicated that several olfactory-related protein of *C. buqueti* showed strong binding affinities to a volatile compound (linalool) from the host plant *N. affinis*, Liu et al. (2023), Yang et al. (2024). Based on the above studies, a total of nine candidate volatiles were selected as test ligands, including eight volatiles emitted by female adults of *C. buqueti* and the linalool released from the host plant *N. affinis* (Table 1). All these compounds were purchased from Aladdin Biochemical Technology Co., LTD. (Shanghai, China) with a purity of at least more than 90%. The binding affinities of various volatile ligands to CbuqOBP1 was assessed by adding each competitor ligand at different concentrations with both CbuqOBP1 and 1-NPN at a concentration of 2 μM. Dissociation constants (*K*_i_) of competitors to CbuqOBP1 were determined based on the corresponding half-maximal inhibitory concentration (IC_50_) values using the formula: *K*_i_ = [IC_50_]/(1 + [1-NPN]/*K*_1–NPN_), where [1-NPN] is the free concentration of 1-NPN and *K*_1–NPN_ represents the *K*_d_ of the CbuqOBP1/1-NPN complex. Binding data were collected as three independent measurements.

**TABLE 1:** Binding affinities of tested volatiles to CbuqOBP1.

Ligand	Structural formula	CAS no.	IC_50_ (μM)	*K*_i_ (μM)
*C. buqueti* volatiles				
Styrene	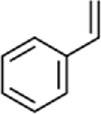	100–42–5	22.07	20.39
(+)-Limonene	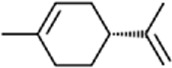	5,989–27–5	18.78	17.35
Benzothiazole	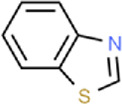	95–16–9	12.03	11.11
M-xylene	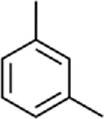	108–38–3	35.99	33.24
Phenol	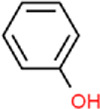	108–95–2	11.36	10.49
Cedrol	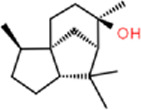	77–53–2	40.25	37.19
trans,trans-2,4-Nonadienal	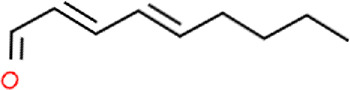	5,910–87–2	24.27	22.42
Ethyl hexanoate	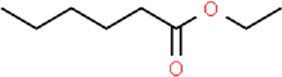	123–66–0	21.05	19.45
Host plant volatiles				
Linalool	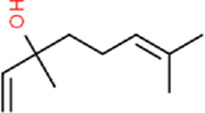	78–70–6	14.52	13.41

### 2.6 Structural modeling and molecular docking

After the signal peptide was removed, the three-dimensional (3D) structure of CbuqOBP1 was simulated using AlphaFold Colab (Jumper et al., 2021) at https://colab.research.google.com/github/deepmind/alphafold/blob/main/notebooks/AlphaFold.ipynb. Ramachandran plots were constructed using the pymod3 plugin implemented in PyMOL v2.4.2 to evaluate the stereochemical quality of the modeled 3D structure. Based on the florescence binding assay, compounds that exhibited an IC_50_ value of less than 15 μM (Table 1) were chosen as candidates for further molecular docking simulations to determine the ligands’ binding mode. The 3D structures of these ligands in SDF format were downloaded from PubChem (https://pubchem.ncbi.nlm.nih.gov/). The molecular docking analysis was conducted utilizing the CB-Dock2 online platform (https://cadd.labshare.cn/cb-dock2/), and the optimal docking model was chosen based on the binding energy with the most negative value (Liu et al., 2022). The interactions between CbuqOBP1 and ligands were analyzed using LigPlot^+^ v.2.2.8. The model visualization was performed using PyMOL v2.4.2.

### 2.7 Statistical analysis

Statistical analysis and graphs in this study were produced using GraphPad Prism v9.4.1. The significance of differences in developmental stage-specific expression was first determined using one-way ANOVA followed by a Tukey’s multiple comparison test, and then the non-paired Student’s t-test was further conducted to compared the statistical difference in gene expression levels between male and female adults. Significant differences of gene expression levels across various tissues between male and female *C. buqueti* were also analyzed by the non-paired Student’s t-test. All data were presented as mean ± standard deviation with three biological replicates, with *P* < 0.05 considered significant.

## 3 Results

### 3.1 Sequence analysis of CbuqOBP1

**FIGURE 1:**
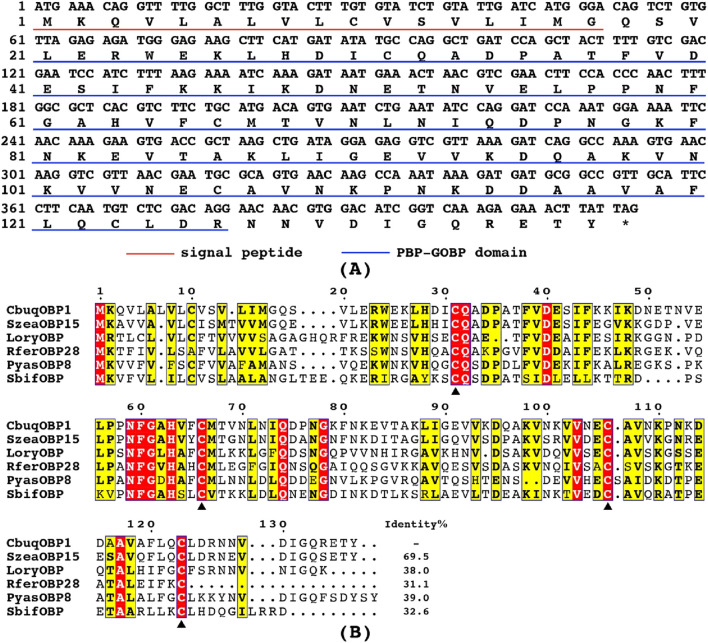
**(A)** Nucleotide and deduced amino acid sequences of the *CbuqOBP1* gene. The signal peptide and the PBP–GOBP domain are underlined in red and blue, respectively. The asterisk represents the stop codon. **(B)** Alignment of amino acid sequences of CbuqOBP1 with the OBPs from other coleopteran insects, including *S. zeamais* (SezaOBP15, QCT83269.1), *Lissorhoptrus oryzophilus* (LoryOBP, AHE13791.1), *R. ferrugineus* (RferOBP28, ATU47278.1), *Pachyrhinus yasumatsui* (PyasOBP8, WJJ63266.1) and *S. bifasciatus* (SbifOBP, UTN00822.1). The conserved cysteines are highlighted by black triangles. Strictly conserved residues are highlighted in white letters with a red background. Black letters with a yellow background represent the 70%–100% conservation in amino acid residues.

As shown in Figure 1A, the full-length ORF of *CbuqOBP1* is composed of 414 nucleotides that encodes a protein of 137 amino acid residues with a 17-residue signal peptide at the N-terminal. CbuqOBP1 also contains one conserved pheromone binding protein-general-odorant binding protein (PBP–GOBP) domain, which is the typical characteristic of insect OBPs. The full-length ORF sequence of *CbuqOBP1* was verified by PCR cloning and sequencing, and was deposited in GenBank under the accession number PQ067317. The predicted molecular weight for CbuqOBP1 is 15.33 kDa with an isoelectric point of 5.38.

The amino acid sequence alignment of CbuqOBP1 with corresponding OBPs from other beetles revealed that CbuqOBP1 exhibited a maximum identity of 69.5% with SzeaOBP15 from *Sitophilus zeamais*, followed by PyasOBP8 from *Pachyrhinus yasumatsui* (39.0%), LoryOBP from *Lissorhoptrus oryzophilus* (38.0%), SbifOBP from *Semanotus bifasciatus* (32.6%) and RferOBP28 from *R*. *ferrugineus* (31.1%) (Figure 1B). Compared to six conserved cysteines in classical OBPs, CbuqOBP1 contains only four conserved cysteine residues (Figure 1B), indicating that CbuqOBP1 belongs to the Minus-C insect OBP subfamily.

### 3.2 Phylogenetic analysis

**FIGURE 2:**
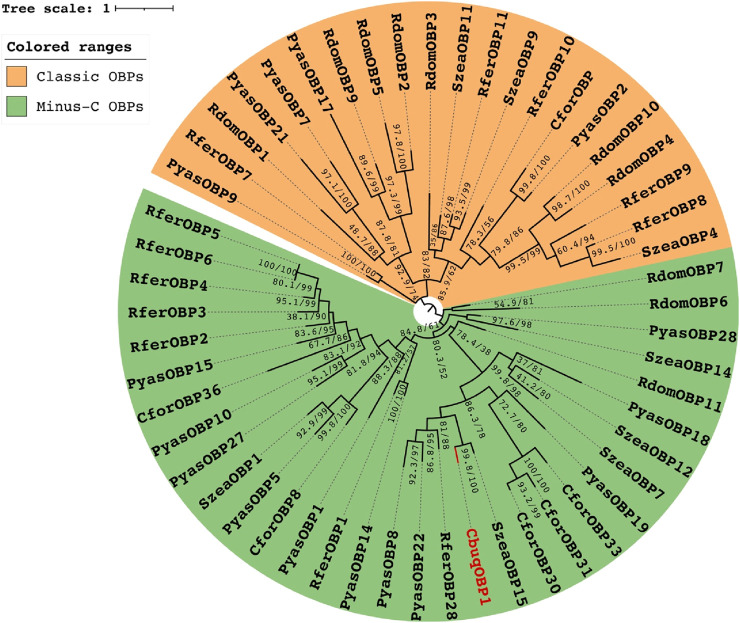
Maximum-likelihood tree of CbuqOBP1 amino acid sequences with OBPs from other species of Coleoptera. CbuqOBP1 is shown in red. Bootstrap support values and SH-aLRT values are indicated on branches. A midpoint approach was applied to root the tree. The insect species include *Pachyrhinus yasumatsui* (Pyas), *R. ferrugineus* (Rfer), *Rhyzopertha dominica* (Rdom), *S. zeamais* (Szea) and *C. formicarius* (Cfor), and GenBank accession numbers are provided in Supplementary Table S2.

To evaluate the evolutionary relationships among proteins, a phylogenetic tree was constructed using the amino acid sequences of CbuqOBP1 and other 52 OBPs from five species of Coleoptera (Figure 2), including *P. yasumatsui* (Hong et al., 2023), *R. ferrugineus* (Yan et al., 2016), *Rhyzopertha dominica* (Mandiana Diakite et al., 2016), *S. zeamais* (Tang et al., 2019) and *Cylas formicarius* (Bin et al., 2017; Hua et al., 2021). The results showed that Minus-C OBPs and Classic OBPs formed two subgroups and CbuqOBP1 fell within the subgroup of Minus-C OBPs. CbuqOBP1 shared the closest evolutionary relationship with SzeaOBP15, which was consistent with the results of multiple sequence alignment.

### 3.3 The spatial-temporal expression profiles of *CbuqOBP1*

**FIGURE 3:**
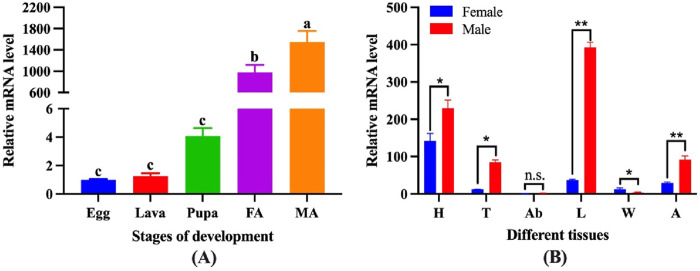
The spatial-temporal expression profiles of *CbuqOBP1*. **(A)** Relative mRNA expression levels of the *CbuqOBP1* at different developmental stages. Different lowercase letters above the bars indicate significant differences among different stages at the *P* value <0.05 level. FA, female adult; MA, male adult. **(B)** Expression profiles analysis of *CbuqOBP1* in various tissues of female and male adults. Statistically significant differences are indicated by * (*P* < 0.05) and ** (*P* < 0.01). The n. s Denotes no significant difference. H, head; T, thorax; Ab, abdomen; L, leg; W, wing; A, antenna.

The expression levels of *CbuqOBP1* at different developmental stages (eggs, larvae, pupae and adults of both sexes) were investigated by qPCR (Figure 3A). The results revealed that the expression of *CbuqOBP1* was developmentally regulated and showed an adult dominant expression profile, with very low transcription levels in egg, larval, and pupal stages. Furthermore, the expression level of *CbuqOBP1* in the adult stage was significantly male-biased, with an expression level approximately 1.6-fold higher in male adults than in female adults (*P* < 0.05, Student’s t-test).

The expression profiles of *CbuqOBP1* in different body parts of both male and female adults were further investigated, including head without antenna, thorax, abdomen, leg, wing and antenna (Figure 3B). The results showed that *CbuqOBP1* had a broad tissue expression profile and exhibited a dominant expression in the leg of male. Additionally, *CbuqOBP1* was also expressed highly in the head of both sexes, the thorax and antenna of the male. In general, the expression level of *CbuqOBP1* in various tissues of male adults is higher than that in female adults.

### 3.4 Expression and purification of CbuqOBP1 protein

**FIGURE 4:**
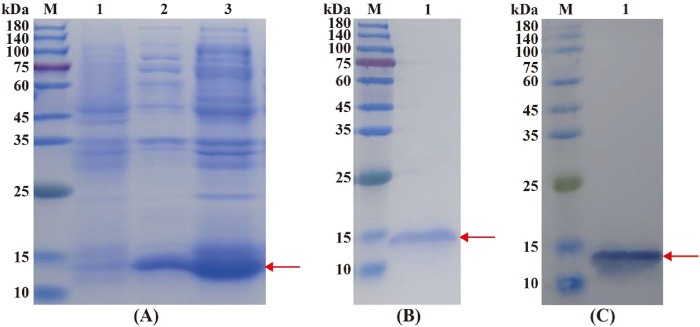
Identification of the recombinant CbuqOBP1 protein. **(A)** SDS-PAGE analysis of the expression of recombinant CbuqOBP1 protein. Lane M, protein molecular weight marker; Lane 1, the non-induced bacterial culture; Lane 2, the soluble supernatant; Lane 3, inclusion bodies. **(B)** Purification of the recombinant CbuqOBP1. Lane M, protein molecular weight marker; Lane 1, purified recombinant protein. **(C)** Verification of the recombinant CbuqOBP1 by Western blot. Lane M, protein molecular weight marker; Lane 1, the blotting band of the recombinant CbuqOBP1 protein. The recombinant CbuqOBP1 are indicated by red arrowheads.

The molecular weight of the recombinant CbuqOBP1 was predicted to be 15.82 kDa. SDS-PAGE displayed a target protein band at about 15 kDa in the supernatant and precipitate, and this band was very close to the theoretical molecular weight of the fusion protein, indicating that CbuqOBP1 protein was expressed in both the supernatant and inclusion bodies (Figure 4A). After the recombinant protein was purified from the supernatant, a single clear band was observed at about 15 kDa (Figure 4B). The size of the recombinant CbuqOBP1 was further tested by Western blot that showed a band of approximately 15 kDa (Figure 4C). Above results suggested that the recombinant CbuqOBP1 protein had been successfully expressed in BL21 competent cells.

### 3.5 Binding characteristic of recombinant CbuqOBP1

**FIGURE 5:**
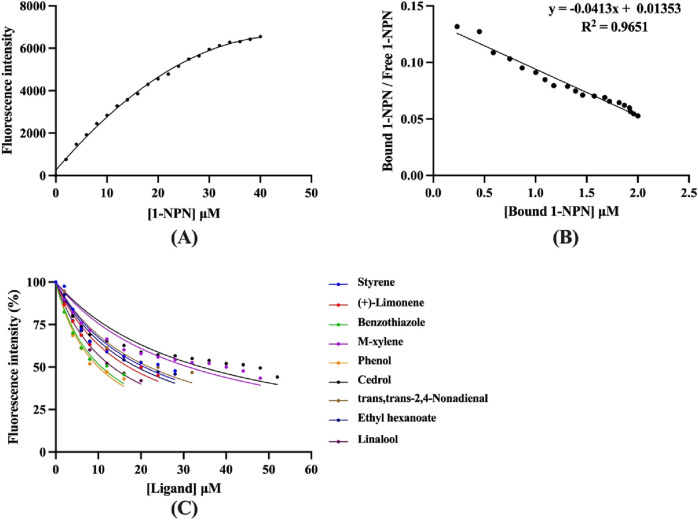
Fluorescence competitive binding assays of CbuqOBP1. **(A,B)** The binding curve and Scatchard plot of 1-NPN to CbuqOBP1. **(C)** Competitive binding curves of CbuqOBP1 to nine test compounds. Data are the means of three independent duplicates.

To explore the function of CbuqOBP1, fluorescence competitive binding assays were carried out to assess the binding affinities of nine compounds to CbuqOBP1, including eight volatiles emitted by female adults of *C. buqueti* and one volatile released from the host plant *N. affinis*. The dissociation constant of CbuqOBP1/1-NPN complex (*K*_1-NPN_) was first determined and the resulting *K*_1-NPN_ value was then used to calculate the *K*_i_ values of test ligands for CbuqOBP1. The binding curve and Scatchard plot showed that the binding of 1-NPN was saturable and there is a single binding site between CbuqOBP1 and 1-NPN, without an apparent allosteric effect (Figures 5A,B). The *K*_1-NPN_ value was measured as 24.21 μM.

Using 1-NPN as a fluorescence probe, competitive binding curves of all examined ligands were determined (Figure 5C) and corresponding IC_50_ and *K*_i_ values were listed in Table 1. The results revealed that all tested ligands reduced the relative fluorescence intensity of the CbuqOBP1/1-NPN mixture to less than half, indicating CbuqOBP1 could bind to all the tested ligands. Among eight volatiles emitted by *C. buqueti*, CbuqOBP1 exhibited high binding affinities to phenol and benzothiazole with *K*_i_ values of 10.49 and 11.11 μM, respectively. The host plant volatile linalool also showed high binding affinity to CbuqOBP1 with a *K*_i_ value of 13.41 μM.

### 3.6 Structure modeling and molecular docking

**FIGURE 6:**
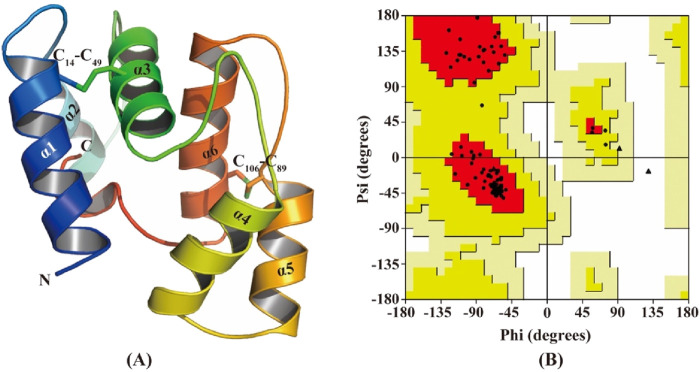
Structural analysis of CbuqOBP1. **(A)** The 3D structure of CbuqOBP1. N and C indicate N-terminus and C-terminus, respectively. Disulfide bonds are indicated by C_14_-C_49_ and C_106_-C_89_. The six α-helices are labeled as α1-α6. **(B)** Ramachandran plot analysis.

The 3D model of CbuqOBP1 was generated using the AlphaFold2-Based Colab server (Figure 6A). The predicted CbuqOBP1 tertiary structure consists of six α helices and is stabilized by two disulfide bridges. These structures constitute a potential binding cavity at the internal of the CbuqOBP1 model. Ramachandran plot analysis revealed that 96.3% of the residues were in the most favored regions, 2.59% were located in the additional allowed regions, and none was trapped in the disallowed region (Figure 6B). Therefore, the stereochemical quality of the predicted 3D structure of CbuqOBP1 is considered reliable and acceptable.

**FIGURE 7:**
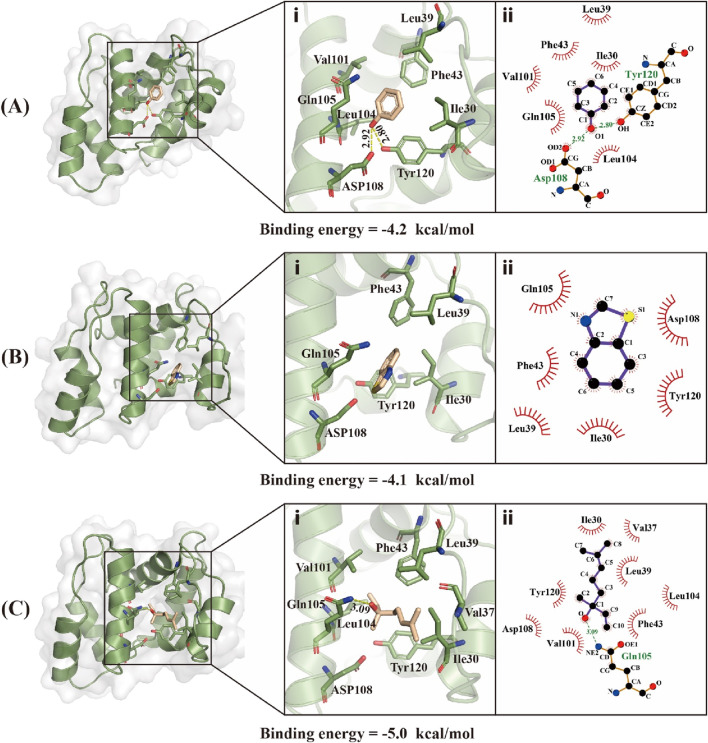
Binding modes of CbuqOBP1 with Phenol **(A)**, Benzothiazole **(B)**, and Linalool **(C)**. (i) 3D demonstrations of the binding interface. (ii) 2D demonstrations of the detailed binding of the key residues with volatile compounds.

Using the predicted structure of CbuqOBP1, the molecular docking simulation was carried out to investigate the binding mechanisms of CbuqOBP1 to three ligands that showed high binding affinity to CbuqOBP1 (phenol, benzothiazole, and Linalool) (Figure 7). Docking results demonstrated that all three ligands bound to the same pocket with the binding energy values of −4.2, −4.1 and −5.0 kcal/mol, respectively. The main interaction between CbuqOBP1 and three ligands primarily relies hydrophobic interactions. Phenol is best docked in a hydrophobic cavity that is composed of Ile30, Leu39, Phe43, Gln105, Asp108 and Tyr120, and Asp108 and Tyr120 are involved in the formation of two hydrogen bonds with the length of 2.92 and 2.80 Å, respectively (Figure 7A). Benzothiazole is best docked in a hydrophobic cavity formed by Ile30, Leu39, Phe43, Gln105, Asp108, and Tyr120 (Figure 7B). Linalool is best docked in a hydrophobic cavity consisting of Ile30, Val37, Leu39, Phe43, Val101, Leu104, Gln105, Asp108 and Tyr120, and a 3.09 Å hydrogen bond is formed between linalool and Gln83 (Figure 7C).

## 4 Discussion

The olfactory perception is related to almost all insect behaviors. The sensitive olfactory system is essential for insects to detect pheromones and plant volatiles (Wang et al., 2024). Acting as the first filter of the olfactory system, the OBP is able to trigger the activation of the target odorant receptors by facilitating the transport of odorants *via* the sensillar lymph and directing them to the dendrites (Pelosi et al., 2006; Leal, 2013). In this study, one OBP gene from *C. buqueti*, *CbuqOBP1*, was identified and functionally characterized. Multiple sequence alignment showed that CbuqOBP1 shares high sequence similarity with other homologous OBPs and possesses four conserved cysteine residues, indicating that CbuqOBP1 belongs to the Minus-C insect OBPs subfamily. This is the first Minus-C OBPs found in *C. buqueti*. Phylogenetic analysis also revealed that CbuqOBP1 was placed into the same branch of Minus-C OBPs from other coleopteran insects, while the Classic OBPs were grouped into another branch. Based on the studies of the origins and evolutionary histories of chemosensory systems, it was postulated that Minus-C OBPs might be the ancestor of the Classic OBPs and the driving force in OBP evolution seems toward introducing more disulfide bridges and more complexity (Vieira and Rozas, 2011; Li Z. Q. et al., 2013).

Analyzing expression patterns of OBPs could offer insights into comprehending the physiological functions of these proteins. Therefore, the qPCR was used to determine the spatial-temporal expression profiles of the *CbuqOBP1*. The results revealed that *CbuqOBP1* was expressed throughout the egg, larval, pupa and adult stages with the significantly high transcription levels in the adult stage, indicating that *CbuqOBP1* plays important roles in chemosensory behavior of adults. A similar developmental expression pattern has been observed in *HarmOBP18*, a Minus-C OBP from the cotton bollworm *Helicoverpa armigera* (Li Z. Q. et al., 2013). In contrast, some Minus-C OBPs might also be related to chemosensory behavior of larvae. For example, *HarmOBP17* from *H. armigera* and *SezaOPB1* from the maize weevil *S. zeamais* exhibited significantly high expression levels in low-instar larvae (Li Z. Q. et al., 2013; Zhang et al., 2019). Numerous studies have indicated that the majority of insect OBPs were predominantly expressed in the antennae of both sexes, suggesting their likely involvement in olfactory processes (Cai et al., 2021; Qu et al., 2021; Yi et al., 2024). On the other hand, the high expression in non-olfactory tissues was also found in a number of OBPs, including heads, thoraxes, abdomen, legs and wings (Yan et al., 2016; Li et al., 2017; Cui et al., 2018; Wang et al., 2021). For example, orthologous *OBP10* from two closely related *Helicoverpa* species in Lepidoptera was expressed in both chemosensory (antennae of both sexes) and reproductive organs (accessory glands and testes of male adults). This OBP is transferred to females during mating and is eventually found on the surface of fertilized eggs. Among the several different volatile compounds present in reproductive organs, OBP10 binds 1-dodecene, a compound reported as an insect repellent. Its affinity with 1-dodecene suggest that OBP10 could be a carrier for oviposition deterrents, favoring spreading of the eggs in these species where cannibalism is active among larvae (Sun et al., 2012). Except for antennae, our results indicated that *CbuqOBP1* exhibited highest expression in the leg of male adults. Highest expression of OBPs in legs has also been reported in various insect species, such as *OBP18* and *OBP23* from *Tomicus* bark beetles (*T. yunnanensis, T. brevipilosus* and *T. minor*) (Lu et al., 2025), and *OBP7* from the oriental fruit fly *Bactrocera dorsalis* (Zheng et al., 2013). These OBPs expressed in legs may participate in taste to perceive non-volatile chemicals or other physiological functions. Further investigation is needed to explore their specific roles. Moreover, it has been reported that expression levels of OBPs may differ between males and females (Yan et al., 2016; Song et al., 2018). *CbuqOBP1* showed the male-biased expression in the spatial-temporal expression profiles, indicating that CbuqOBP1 may be involved in detecting pheromones released by females.

Previous studies have demonstrated the important roles of insect Minus-C OBPs in finding host plants (Li Z. Q. et al., 2013; Zhang F. et al., 2020; Wang et al., 2024). However, little is known about the functions of insect Minus-C OBPs in recognizing sex pheromones or interspecific communication. Therefore, the function of the CbuqOBP1 was further investigated by the ligand binding assays with nine volatile compounds, including eight emitted by *C. buqueti* and one released from the host plant (*N. affinis*). The binding characteristics of two PBPs from *C. buqueti* with above volatile compounds were also tested in our previous studies (Yang et al., 2017b; Liu et al., 2023). Different from the binding characteristics of two PBPs from *C. buqueti*, CbuqOBP1 possessed a broader ligand spectrum and showed strong binding affinity with phenol and benzothiazole among 8 *C. buqueti* volatiles. It has been reported that benzothiazole were highly attractive to *Bradysia odoriphaga* (Diptera) and *Xylotrechus rusticus* (Coleoptera) adults (Li L. et al., 2013; Chen et al., 2014), and phenol is a major component of *Costelytra zealandica* (Coleoptera) sex pheromone (Meng et al., 1999). Previous behavioral studies demonstrated that phenol and benzothiazole was efficient attractants for *C. buqueti* adults (Cai, 2018). Therefore, CbuqOBP1 most likely participates in the process of detecting and transporting sex pheromones, but further study is needed to confirm this. In addition, CbuqOBP1 also showed high affinity with the host volatile (linalool), suggesting its likely involvement in *C. buqueti* chemoreception of host plants.

To further understand the molecular interactions between CbuqOBP1 and its ligands, the 3D structure model of CbuqOBP1 was constructed and the binding mode of CbuqOBP1 to phenol, benzothiazole and linalool was determined *via* molecular docking analysis. The predicted CbuqOBP1 tertiary structure possesses only two disulfide bridges and an internal cavity, which is similar to that reported for *Monochamus alternatus* (Coleoptera) OBP1 (Zhang F. et al., 2020), *A. mellifera* (Hymenoptera) OBP14 (Spinelli et al., 2012) and *D. helophoroides* (Coleoptera) OBP21 (Li et al., 2015). It has been reported that the loss of one disulfide bridge in Minus-C OBPs could result in a more flexible structure (Li et al., 2015). The addition of the new disulfide bridge caused reduced flexibility, thereby influencing the capacity of the binding pocket to accommodate various odorants (Spinelli et al., 2012; Zhuang et al., 2014). The conformational flexibility of *Dastarcus helophoroides* OBP21 affected both the ligand binding range and the binding affinity for specific ligands (Li et al., 2015). This may be the reason why CbuqOBP1 exhibits broad binding capabilities. The hydrophobic interactions between insect OBPs and their ligands have been demonstrated by numerous studies and hydrophobic interactions are essential for the specificity of ligand binding, such as *Apolygus lucorum* (Hemiptera) OBP22 (Liu et al., 2019). The existence of hydrophobic forces contributes to reducing the exposure of the protein to water, thereby ensuring its stable conformation and proper function (Zhuang et al., 2014). In our study, binding models of CbuqOBP1 to three ligands also indicated that hydrophobic interactions were the dominant forces within the binding cavities of CbuqOBP1. Hydrogen bonds were also involved in the interaction of CbuqOBP1 with phenol and Linalool, which was able to promote binding. It is noteworthy that Ile30, Leu39, Phe43, Gln105, Asp108 and Tyr120 were highly overlapped and were all involved in the binding process with three ligands by the hydrophobic interactions or contributing hydrogen bonds, suggesting that these residues may be the key binding sites for CbuqOBP1 and play key roles in odorant-binding.

## 5 Conclusion

In summary, the first Minus-C OBP of *C. buqueti* was functionally characterized. The results presented in this work revealed significant differences in the expression levels of *CbuqOBP1* across various developmental stages, tissues and genders. Similar to some PBPs, *CbuqOBP1* showed the male-biased expression in the spatial-temporal expression profiles. Binding assays and docking analysis further revealed the dual roles of CbuqOBP1 in binding volatile compounds from the host plant and the same species, suggesting another potential function of CbuqOBP1 to bind sex pheromone. However, further experiments would be required to validate the physiological functions of CbuqOBP1, such as gene knockdown studies, site-directed mutagenesis and corresponding behavioral experiments. The findings of this study will aid in further functional investigations of the olfactory recognition mechanism of *C. buqueti* and provide a theoretical basis for developing novel control strategies to help mitigating the damage of this pest.

## Data Availability

The original contributions presented in the study are included in the article/Supplementary Material, further inquiries can be directed to the corresponding author.
