# Supplementary material for: Molecular characterization of a Minus-C odorant-binding protein from Cyrtotrachelus buqueti (Coleoptera: Curculionidae)
Source: Front Physiol. 2025 Apr 25;16:1586738. doi: 10.3389/fphys.2025.1586738 (PMC12061717; doi:10.3389/fphys.2025.1586738)
Supplement: Supplementary file 1 [file Table1.docx]

**Table S1.** Primer sequences used in this study.

| **Primer name** | **Sequences (5′-3′) *^a^*** |
| --- | --- |
| Primers for qPCR | |
| qOBP1-F | ACAGTCTGTGTTAGAGAGATGGGAG |
| qOBP1-R | TCTCCTATCAGCTTAGCGGTCAC |
| qGAPDH-F | TGACCGTCAGATTGAAGAAAGC |
| qGAPDH-R | ACACCCTTCAACTCACCCTCG |
| Primers for prokaryotic expression | |
| OBP1-F | GTGCCGCGCGGCAGC**CATATG**CAGTCTGTGTTAGAGAGATGGGAGAA |
| OBP1-R | GTGGTGGTGGTGGTG**CTCGAG**CTAATAAGTTTCTCTTTGACCGATGTC |

*^a^* The sites for restriction digest in primers for prokaryotic expression are indicated in bold.
