# Supplementary material for: Molecular characterization of a Minus-C odorant-binding protein from Cyrtotrachelus buqueti (Coleoptera: Curculionidae)
Source: Front Physiol. 2025 Apr 25;16:1586738. doi: 10.3389/fphys.2025.1586738 (PMC12061717; doi:10.3389/fphys.2025.1586738)
Supplement: Supplementary file 2 [file Table2.docx]

**Table S2.** GenBank accession numbers of OBP sequences from other species of Coleoptera.

| **Species** | **Protein name** | **GenBank accession No.** |
| --- | --- | --- |
| *Rhyzopertha dominica* | RdomOBP1 | AIX97124.1 |
|  | RdomOBP2 | AIX97125.1 |
|  | RdomOBP3 | AIX97126.1 |
|  | RdomOBP4 | AIX97127.1 |
|  | RdomOBP5 | AIX97128.1 |
|  | RdomOBP6 | AIX97129.1 |
|  | RdomOBP7 | AIX97130.1 |
|  | RdomOBP9 | AIX97132.1 |
|  | RdomOBP10 | AIX97133.1 |
|  | RdomOBP11 | AIX97134.1 |
| *Sitophilus zeamais* | SzeaOBP1 | QCT83255.1 |
|  | SzeaOBP4 | QCT83258.1 |
|  | SzeaOBP7 | QCT83261.1 |
|  | SzeaOBP9 | QCT83263.1 |
|  | SzeaOBP11 | QCT83265.1 |
|  | SzeaOBP12 | QCT83266.1 |
|  | SzeaOBP14 | QCT83268.1 |
|  | SzeaOBP15 | QCT83269.1 |
| *Rhynchophorus ferrugineus* | RferOBP1 | ANE37545.1 |
|  | RferOBP2 | ANE37546.1 |
|  | RferOBP3 | ANE37547.1 |
|  | RferOBP4 | ANE37548.1 |
|  | RferOBP5 | ANE37549.1 |
|  | RferOBP6 | ANE37550.1 |
|  | RferOBP7 | ANE37551.1 |
|  | RferOBP8 | ANE37552.1 |
|  | RferOBP9 | ANE37553.1 |
|  | RferOBP10 | ANE37554.1 |
|  | RferOBP11 | ANE37555.1 |
|  | RferOBP28 | ATU47278.1 |
| *Pachyrhinus yasumatsui* | PyasOBP1 | WJJ63261.1 |
|  | PyasOBP2 | UUK33715.1 |
|  | PyasOBP5 | UUK33716.1 |
|  | PyasOBP7 | WJJ63265.1 |
|  | PyasOBP8 | WJJ63266.1 |
|  | PyasOBP9 | WJJ63267.1 |
|  | PyasOBP10 | WJJ63268.1 |
|  | PyasOBP14 | WJJ63272.1 |
|  | PyasOBP15 | WJJ63273.1 |
|  | PyasOBP17 | WJJ63275.1 |
|  | PyasOBP18 | WJJ63276.1 |
|  | PyasOBP19 | WJJ63277.1 |
|  | PyasOBP21 | WJJ63279.1 |
|  | PyasOBP22 | WJJ63280.1 |
|  | PyasOBP27 | WJJ63285.1 |
|  | PyasOBP28 | WJJ63286.1 |
| *Cylas formicarius* | CforOBP | QFO46787.1 |
|  | CforOBP8 | QBI90150.1 |
|  | CforOBP30 | UNA06114.1 |
|  | CforOBP31 | UNA06115.1 |
|  | CforOBP33 | UNA06117.1 |
|  | CforOBP36 | UZC53364.1 |
